# Habitat use, movement and activity of two large‐bodied native riverine fishes in a regulated lowland weir pool

**DOI:** 10.1111/jfb.14275

**Published:** 2020-02-23

**Authors:** Wayne M. Koster, David R. Dawson, Adrian Kitchingman, Paul D. Moloney, Robin Hale

**Affiliations:** ^1^ Department of Environment, Land, Water and Planning Arthur Rylah Institute for Environmental Research Heidelberg Australia

**Keywords:** accelerometer, acoustic telemetry, native fish conservation, river regulation

## Abstract

The construction of dams and weirs, and associated changes to hydrological and hydraulic (*e.g.*, water level and velocity) characteristics of rivers is a key environmental threat for fish. These multiple stressors potentially can affect fish in a variety of ways, including by causing changes in their movement, habitat use and activity. Understanding how and why these changes occur can inform management efforts to ameliorate these threats. In this context, we used acoustic telemetry to examine habitat use, longitudinal movement and activity of two lowland river fishes, Murray cod *Maccullochella peelii* and golden perch *Macquaria ambigua*, in a weir pool environment in south‐eastern Australia. We compared our results to published studies on riverine populations to determine if their behaviours are similar (or not). We show that *M. peelii* and *M. ambigua* in a weir pool exhibited some similar behaviours to conspecific riverine populations, such as strong site fidelity and use of woody habitat for *M. ambigua*. However, some behaviours, such as large‐scale (tens–hundreds of kilometres) movements documented for riverine populations, were rarely observed. These differences potentially reflect flow regulation (*e.g.*, stable water levels, loss of hydraulic cues) in the weir pool. The two species also exhibited contrasting responses to dissolved oxygen conditions in the weir pool, which may reflect differences in their life history. Overall, this study shows that although some aspects of these two native fishes' life history can continue despite flow regulation, other aspects may change in weir pools, potentially impacting on long‐term population persistence.

## 1 INTRODUCTION

The construction of dams and weirs to store or divert water for human use has altered the condition of aquatic ecosystems worldwide (Dudgeon *et al*., 2006; Jo *et al*., 2019; Olden, 2016; Vörösmarty *et al*., 2010). Major alterations include reduced flow variability, conversion of lotic to lentic habitats, reduced habitat complexity and altered water quality (Bunn & Arthington, 2002). These changes impact ecological processes and have effects on a range of biota, including fish, invertebrates and plants. For instance, conversion of lotic habitat to lentic in rivers due to the impoundment of upstream waters in dams and weirs, and associated changes to flow variability, water velocity, level and turbulence have been associated with the elimination of pelagic spawning fishes such as burbot *Lota lota* (Linnaeus 1758) in the Great River Ouse, UK (Copp, 1990), extinctions of aquatic snails in the lower River Murray, Australia (Sheldon & Walker, 1997), and decreased wood decomposition in regulated Mediterranean rivers (Abril *et al*., 2015).

Flow regulation can affect fish in a variety of ways, across cellular, individual, population and community levels (Murchie *et al*., 2008). However, many animals, including fish, alter their behaviour as an initial response to environmental change and this can be a strong determinant of fitness outcomes (Wong & Candolin, 2015). Consequently, behavioural responses of fish (*e.g*., habitat use, movement, activity) to flow regulation are particularly important. For example, some fish shift position during periods of flow alteration or alter their habitat use or feeding, and these behaviours have implications for growth, condition and reproduction (Alexandre *et al*., 2016; Brenden *et al*., 2006; Del Mar Torralva *et al*., 1997). Understanding these changes to behaviour is critical therefore to evaluate impacts of alterations due to regulation and to guide management actions.

In Australia, regulation of river systems with dams and weirs has been extensive. In the Murray–Darling Basin (MDB), Australia's largest drainage basin, more than 4000 licensed in‐stream structures have been constructed to regulate river flow (Lintermans, 2007). Along the main river in the MDB, the Murray River, weirs have created a series of contiguous pools for at least 700 km in the lower reaches (Walker, 2006). This modification has been identified as a driver of severe declines of biota, such as extinctions of native riverine fish species, including trout cod *Maccullochella macquariensis* (Cuvier 1829) and Macquarie perch *Macquaria australasica* (Cuvier 1830), and declines in other species such as silver perch *Bidyanus bidyanus* (Mitchell 1838) in the lower Murray River (Mallen‐Cooper & Zampatti, 2018; Wedderburn *et al*., 2017). Elsewhere in the MDB dramatic fish kills have occurred from low dissolved oxygen concentrations (hypoxia) during reduced flows, such as in Rices Weir on Broken Creek and the Menindee Weir on the Darling River, with the latter attracting global media attention (Normile, 2019; Stewardson & Skinner, 2018).

Murray cod *Maccullochella peelii* (Mitchell 1838) and golden perch *Macquaria ambigua* (Richardson 1845) are two large native riverine fish species of recreational angling and conservation significance endemic to south‐eastern Australia. Populations of both species have declined substantially in the MDB due to factors such as habitat degradation and loss, altered flow regimes, reduced water quality and barriers to movement (Koehn & Nicol, 2014; Leigh & Zampatti, 2013; Thiem *et al*., 2017; Wedderburn *et al*., 2017). *M. peelii* is listed as Threatened nationally and *M. ambigua* as Near Threatened in Victoria. The two species display distinct life history strategies and thus may behave differently in response to environmental changes associated with river flow regulation, such as loss of hydraulic cues, altered water quality or reduced habitat complexity in weir pools.

Studies on riverine populations have shown that *M. ambigua* spawn and migrate in response to increases in flow or water level (Koster *et al*., 2017; Reynolds, 1983) whereas *M. peelii* spawning and migration is not dependent on flow increases (Humphries *et al*., 1999; Koehn *et al*., 2009). *M. peelii* has a greater sensitivity to hypoxia than *M. ambigua* (Small *et al*., 2014) and there is evidence that *M. ambigua* may be more likely to move away from areas affected by hypoxic conditions than *M. peelii* (King *et al*., 2012; Koster *et al*., 2014b; Leigh & Zampatti, 2013). Both species use woody habitat, but *M. peelii* use deeper habitats, with higher water velocities, than *M. ambigua* (Koehn & Nicol, 2014). Given that environmental conditions such as hydraulics, water quality or habitat might be altered in weir pools, it is important to understand if fishes exhibit similar behaviour in weirs compared to rivers.

The aim of this study was to quantify daily and seasonal habitat use patterns and the longitudinal movement and activity of *M. peelii* and *M. ambigua* in relation to environmental conditions in a weir pool environment. We used a fine‐scale continuous acoustic telemetry array, integrated with side‐scanning sonar, to assess instream habitat conditions to (a) characterize the behaviour of fish in weir pools and evaluate if this is similar to knowledge about the traits and behaviours of these species in riverine populations; and (b) evaluate whether patterns differ among these two species. Knowledge of aspects of life history such as habitat use, movement and activity in weir pools is needed to better understand potential impacts of habitat change and flow regulation on these two species, and to inform potential management interventions. This information will also potentially inform efforts to assess and mitigate future impacts on other riverine fishes experiencing the effects of flow regulation.

## 2 MATERIALS AND METHODS

### 2.1 Study area

**Figure 1:**
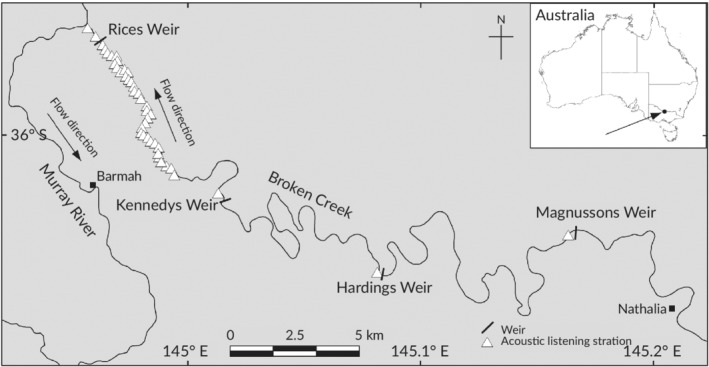
Map showing location of the study area

The study was conducted in lower Broken Creek, south‐eastern Australia (Figure 1). Lower Broken Creek is typically 40–50 m wide and 2–3 m deep. Average daily discharge in Broken Creek is 230 ml (Stewardson & Skinner, 2018). Broken Creek was subject to extensive historical removal of fallen trees and dredging (Trueman, 2011). Under natural conditions Broken Creek flowed intermittently, predominantly during winter–spring, and during most summers contracted to a series of pools (Reich *et al*., 2010). For the past 100 years a series of predominantly contiguous low‐level weirs along lower Broken Creek and diversion channels has maintained permanent water for irrigation (Reich *et al*., 2010). These weirs are similar to those found in many other regulated rivers, that is, low‐level (<3–5 m), narrow (<50–100 m wide) and shallow (<several metres deep) weirs operated to maintain a steady upstream pool level for irrigation (Baumgartner, 2007; Baumgartner *et al*., 2014; O'Connor *et al*., 2006). All of the weirs along lower Broken Creek have fishways installed capable of facilitating fish passage (O'Connor *et al*., 2006). Rices Weir, the most downstream weir on Broken Creek, exhibits water‐quality issues, especially low dissolved oxygen concentrations (Stewardson & Skinner, 2018). Major fish kills have become a serious problem in lower Broken Creek in recent years, particularly at Rices Weir. The most likely explanation for the fish kills is low dissolved oxygen levels in conjunction with little or no flow (Rees, 2006).

### 2.2 Acoustic tracking

Twenty‐three *M. peelii* [mean total length (TL) 480 mm, range 320–780 mm; mean weight 2562 g, range 410–10,000 g] and 15 *M. ambigua* (mean TL 351 mm, range 314–415 mm; mean weight 800 g, range 523–1408 g) collected from the lower 5 km of the Rices Weir pool on lower Broken Creek were tagged with acoustic transmitters (Vemco, Nova Scotia, Canada) in August 2012 (Table 1). The transmitter‐to‐fish weight ratio was below about 2% as recommended by Winter (1996). The fish were collected using a Smith‐Root model (Vancouver, Washington, USA) 7.5 GPP boat‐mounted electrofishing unit (500–1000 V, 120 pulses s^−1^).

**Table 1:** Details of the tagged *M. peelii* and *M. ambigua* during the study

Species	ID	TL (mm)	Mass (g)	Total linear range (km)
*M. peelii*	MC1	404	967	1.8
	MC2	407	1162	1.2
	MC3	702	6785	10.0
	MC4	425	1122	2.6
	MC5	698	6381	7.4
	MC6	470	1807	2.2
	MC7	320	422	0.4
	MC8	366	692	0.6
	MC9	390	799	1.2
	MC10	320	410	0.8
	MC11	325	440	0.2
	MC12	344	559	0.8
	MC13	360	645	0.2
	MC14	350	536	0.6
	MC15	710	6255	5.2
	MC16	650	5184	47.1
	MC17	390	876	1.6
	MC18	780	10,000	7.3
	MC19	550	2688	3.5
	MC20	710	7083	3.4
	MC21	440	1111	5.4
	MC22	410	975	5.6
	MC23	510	2022	2.1
*M. ambigua*	GP1	360	782	6.0
	GP2	354	855	1.2
	GP3	340	730	11.2
	GP4	400	1228	1.2
	GP5	314	523	3.0
	GP6	379	994	3.4
	GP7	321	565	2.0
	GP8	350	808	2.0
	GP9	357	802	2.6
	GP10	348	702	48.7
	GP11	330	636	6.0
	GP12	340	664	3.8
	GP13	340	719	0.8
	GP14	320	585	26.9
	GP15	415	1408	4.0

*Note*: ID, identification number; TL, total length.

Vemco V9 accelerometer transmitters (43 × 9 mm, frequency 69 kHz, mass 6.1 g in air, estimated battery life 350 days) were used for *M. ambigua*. Vemco V9 or V13 accelerometer transmitters (42 × 13 mm, frequency 69 kHz, mass 12.2 g in air, estimated battery life 740 days) were used for *M. peelii*, depending on the size of the fish. For both transmitter types the average delay between transmission was 240 s. Acceleration was sampled at 5 Hz for 30 s. The tag calculates a value that represents the root mean square acceleration that results from the combination of the acceleration from each of the three axes averaged over time.

For transmitter implantation, fish were transferred into an aerated, 50 l holding container of river water (temperature 11–13°C) and individually anaesthetized (0.03 ml AQUI‐S per litre water) (AQUI‐S, Lower Hutt, New Zealand). Time to anaesthesia was about 6–9 min. Acoustic transmitters were implanted into the peritoneal cavity through an incision of about 20 mm on the ventral surface, between the pelvic and anal fins. The incision was closed with two dissolvable external synthetic absorbable monofilament sutures. The sex of the fish could not be determined at the time of tagging. Throughout the procedure the head and gills of fish were immersed in aerated anaesthetic solution. Each surgery took about 3 min and the fish was then placed into a recovery net positioned in the creek. Once the fish were observed to maintain their balance and freely swim throughout the net they were subsequently released near the point of capture.

Forty‐two acoustic listening stations (Model VR2W, Vemco) were deployed in Broken Creek between the River Murray junction and Nathalia (a distance of about 50 km) (Figure 1). Thirty‐seven of these listening stations were between Rices Weir and 7 km upstream at about 200 m intervals to provide precise information on fish locations within these reaches (Figure 1). The listening stations were deployed using a length of plastic‐coated steel cable anchored to a log. A float and weight were attached above and below each listening station, respectively, to maintain a vertical position. Each listening station was suspended about 0.5 m above the riverbed. *In situ* tests showed that the listening stations had total detection ranges of about 200 m (*i.e*., 100 m upstream, 100 m downstream), depending on the physical attributes of the site (*e.g*., depth, turbulence). Data were downloaded from the listening stations every 3 months throughout the study.

### 2.3 Habitat mapping

Instream woody habitat (IWH) and stream depth were measured in February 2015 between Rices Weir and 7 km upstream. Locations of IWH masses, defined as individuals and piles of logs and trees with a minimum diameter of 0.1 m and a length of 1 m (excludes small twigs and floating debris), along the stream were recorded using a Trimble GeoExplorer XT6000 series handheld Global Navigation Satellite System (Ultimate Positioning Group Australia, Melbourne, Victoria, Australia) coupled with a laser range finder. Submerged IWH was identified using a Humminbird 998c SI side imaging system (Humminbird Australia, BLA Distribution, Brisbane, Queensland, Australia). At each IWH mass, size [*i.e*., footprint area (m^2^)] and complexity (number of contiguous pieces/large branches) were measured and converted to volume (m^3^ of wood) as per Kitchingman *et al*. (2013). These data were then summarized into total volume (m^3^ of wood) within 100 m upstream and downstream of each logger position. Counts of IWH masses within 100 m upstream and downstream of each logger position were also calculated. The volume of wood in Rices Weir was ~2.2 m^3^, which is similar to much of the entire lower Broken Creek, which comprises predominantly weir pools (A. Kitchingman, unpublished data). Stream depths were recorded using the Hummingbird 998cx SI side scan sonar. These data were summarized into maximum depth (m) within 100 m upstream and downstream of each logger position.

### 2.4 Data analysis

#### 2.4.1 Habitat use

Generalized additive mixed models (Hastie & Tibshirani, 1995) were used to examine patterns of daily and seasonal habitat use, using the mgcv (Wood, 2017) package in R (http://www.r-project.org). Habitat variables examined were (a) IWH volume (m^3^ of wood); (b) count of IWH masses; and (c) maximum stream depth (m). These variables were selected because they are likely important for *M. peelii* and *M. ambigua* (Crook *et al*., 2001; Koehn & Nicol, 2014). Cyclic cubic regression splines (Wood, 2017) were used to smooth the response to hour‐of‐day and day‐of‐year. Individual fish were treated as a random effect. Any autocorrelation was assumed to be autoregressive with order 1 (AR1). Mean values for each habitat variable per hour were calculated for each individual across the entire monitoring period, when transmitters were detected within the intensive listening station array (88% of the time).

#### 2.4.2 Longitudinal movement

Generalized additive mixed models were used to explore relationships between the distances moved by *M. peelii* and *M. ambigua* and environmental variables, using the package mgcv in R. Explanatory variables examined were (a) average daily discharge; (b) change (*t*_1_ − *t*_0_) in daily flow; (c) average daily dissolved oxygen; (d) change (*t*_1_ − *t*_0_) in daily dissolved oxygen; (e) average daily water temperature; (f) diel period; (g) previous movement; (h) day‐of‐year; and (i) water level. Cubic regression splines (Royston & Sauerbrei, 2007) were used to smooth the response to these variables. Individual fish were treated as a random effect. Again, autocorrelations were assumed to be AR1. The model used a log‐normal distribution. Distance moved (km) was calculated as the difference in mean daily position from the current day to the previous day (*t*_1_ − *t*_0_), with position defined as the distance of the receiver from Rices Weir. Total linear ranges for each fish were estimated by determining the distance between the most upstream and downstream locations in Broken Creek.

#### 2.4.3 Activity

Generalized additive mixed models were used to examine relationships between environmental variables and activity (measured in terms of m s^−2^) of *M. peelii* and *M. ambigua*, using the mgcv package in R. Explanatory variables examined were (a) average hourly discharge; (b) change (*t*_1_ − *t*_0_) in hourly flow; (c) average hourly dissolved oxygen; (d) change (*t*_1_ − *t*_0_) in hourly dissolved oxygen; (e) average hourly water temperature; (f) diel period; (g) previous hourly activity; (h) day‐of‐year; and (i) water level. Cubic regression smoothers for water temperature, water level and day‐of‐year were included to account for the possibility of peak activity. Mean values of activity (m s^−2^) per hour were calculated for each individual across the entire monitoring period.

## 3 RESULTS

### 3.1 Discharge and water quality

**Figure 2:**
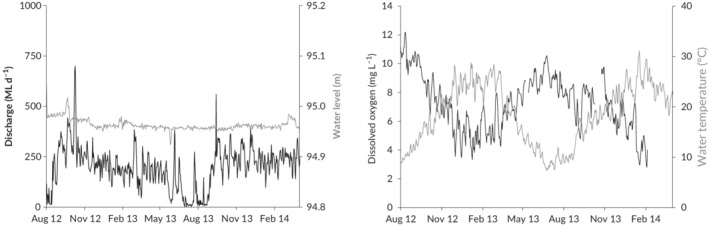
Discharge (black line) and water level (grey line) (left) and dissolved oxygen (black line) and water temperature (grey line) (right) in Broken Creek at Rices Weir throughout the study period

Discharge, water level, water temperature and dissolved oxygen data were obtained from the gauging station at Rices Weir. Discharge was typically lowest around June–August (~50–100 ml day^−1^) and highest around September–October (about 600–700 ml day^−1^) in each year (Figure 2). Several increased discharge events driven by rainfall runoff from the catchment occurred during the study. Water level remained stable throughout most of the study period (Figure 2). Dissolved oxygen generally remained above 4 mg l^−1^ over the study period, although there were short periods (<1–2 days) when dissolved oxygen decreased to about 3 mg l^−1^ between late November 2012 and early February 2013, and between late January and early February 2014 (Figure 2). Maximum temperatures of around 33°C were reached in January and minimum temperatures of around 7°C occurred in June (Figure 2).

### 3.2 Habitat use

**Figure 3:**
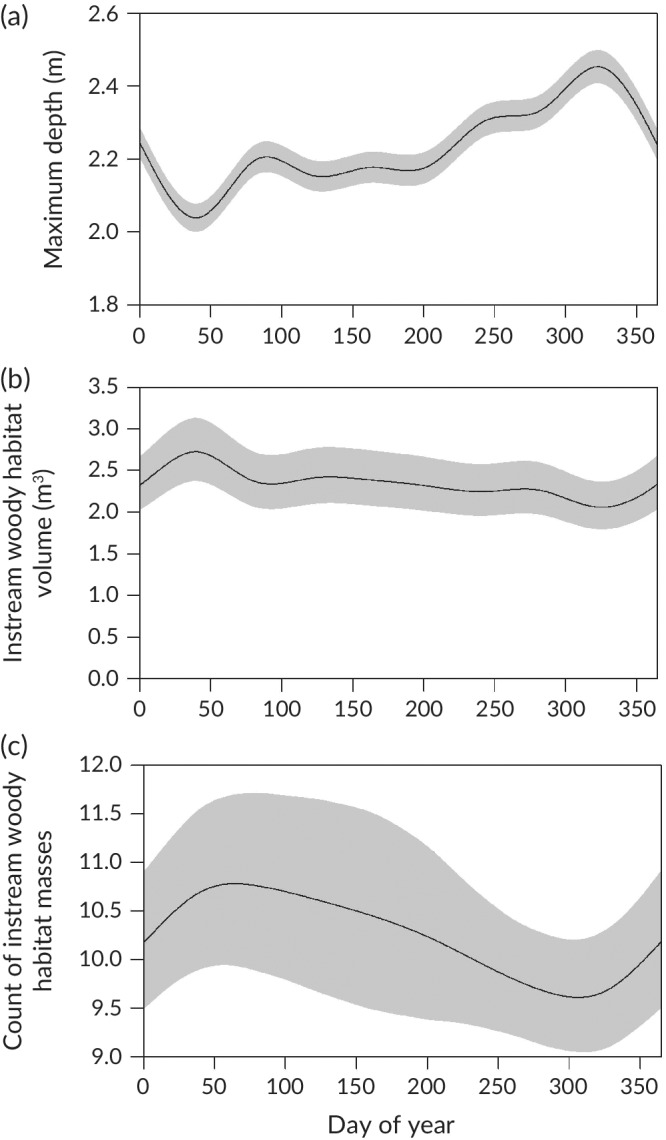
Modelled habitat use of *M. ambigua* throughout the year. Grey shading denotes 95% credible interval

**Figure 4:**
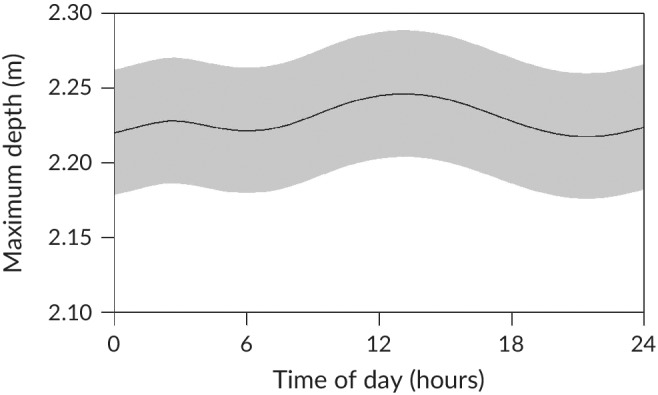
Modelled habitat use of *M. ambigua* throughout day and night. Grey shading denotes 95% credible interval

All fish (23 *M. peelii* and 15 *M. ambigua*) were detected by the listening stations. Habitat use of *M. ambigua* varied throughout the year (Figure 3). Fish were more likely to occupy deeper habitats from October to December, and shallower water from January to February (Figure 3a). They were also more likely to use areas with greater IWH volume and counts of IWH masses around February, and areas with lower counts of IWH masses around November (Figure 3b,c). *M. ambigua* were also more likely to be in deeper areas during the day, particularly from around midday to early afternoon, although the ecological significance of this result is questionable given a very small effect size (Figure 4). The generalised additive mixed models indicated no significant patterns of daily and seasonal habitat use for *M. peelii*.

### 3.3 Longitudinal movement

**Figure 5:**
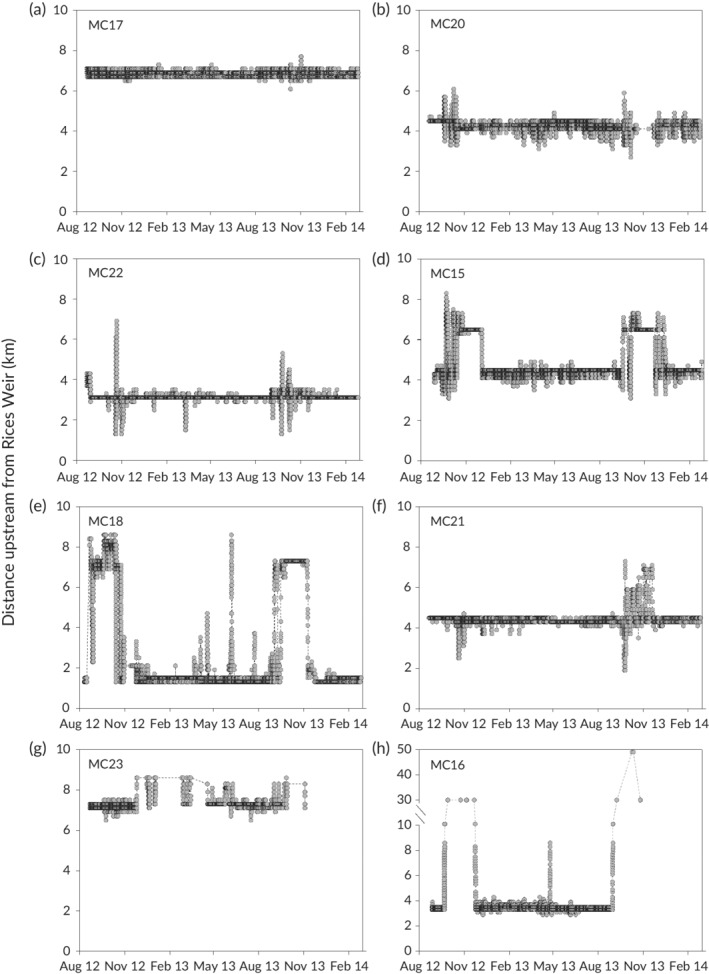
Examples of movement patterns of *M. peelii* tagged in Broken Creek. Grey circles show detections of tagged fish on the listening stations

*M. peelii* typically occupied short stretches of stream (median total linear range 2.1 km) (Figure 5). About one‐third (7 out of 23) of the *M. peelii* (MC3, 702 mm TL; MC5, 698 mm TL; MC15, 710 mm TL; MC16, 650 mm TL; MC18, 780 mm TL; MC 21, 440 mm TL; MC22, 410 mm TL) occasionally moved upstream or downstream (*i.e*., mostly about 5–10 km) from their usual locations (Figure 5). Most (6 out of 7) of these fish returned to the area they had previously occupied; some return movements occurred within 1–2 days (*e.g*., Figure 5c), while others occurred 4–5 weeks later (*e.g*., Figure 5d). This pattern of behaviour was most common in September–November. The median size of the individuals that moved was 698 mm, compared to 390 mm for those that did not move. The generalised additive mixed models indicated no significant relationships between the distances moved by *M. peelii* and environmental variables.

**Figure 6:**
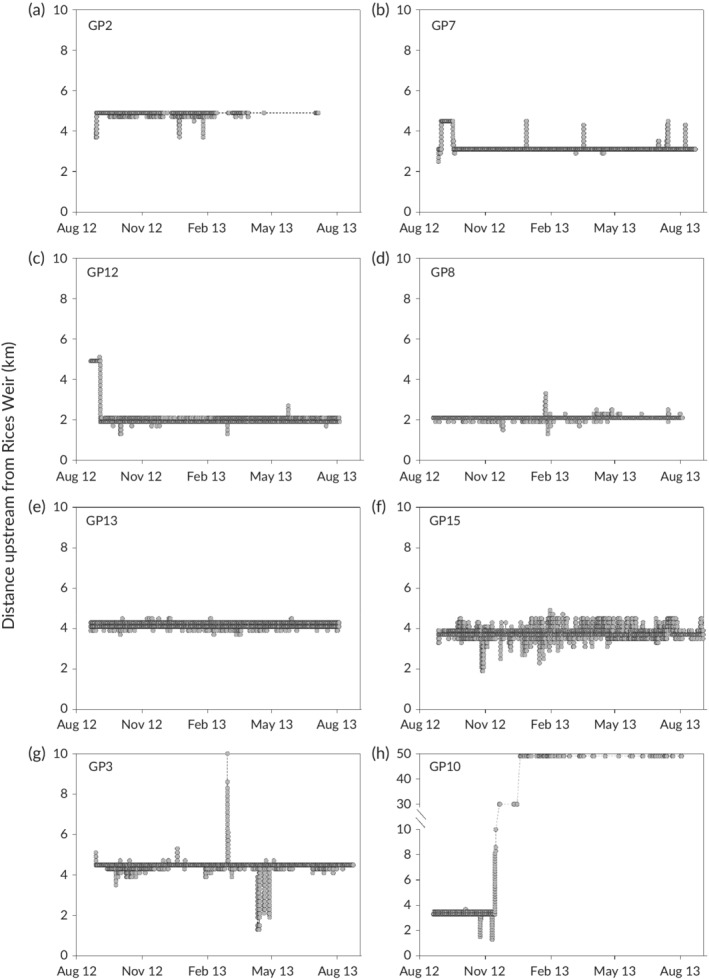
Examples of movement patterns of *M. ambigua* tagged in Broken Creek. Grey circles show detections of tagged fish on the listening stations

Similar to *M. peelii*, *M. ambigua* typically occupied short stretches of stream (median total linear range 3.4 km) (Figure 6). Two of the *M. ambigua* (GP10, 348 mm TL; GP14, 320 mm TL) moved upstream long distances (*i.e*., 25–50 km) in November–December away from their usual locations (*e.g*., Figure 6h). Two *M. ambigua* also moved a short distance downstream into the Murray River in September and January and did not return to Broken Creek. The generalised additive mixed models indicated no significant relationships between the distances moved by *M. ambigua* and environmental variables.

### 3.4 Activity

**Figure 7:**
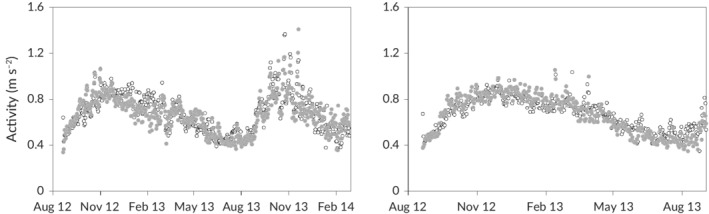
Daily mean activity patterns of *M. peelii* (left) and *M. ambigua* (right) in Broken Creek throughout the study period. Grey circles represent night and white circles represent day

*M. peelii* and *M. ambigua* activity tended to gradually increase from August through to a peak in November–December and then decline or plateau to February (Figure 7). Activity in both species was strongly influenced by previous activity and change in dissolved oxygen (Table 2). Decreases in dissolved oxygen were associated with decreased activity for *M. peelii* and increased activity for *M. ambigua*. As an example, if dissolved oxygen decreased by 10%, activity decreased by 4.1% in *M. peelii* and increased by 5.3% in *M. ambigua*. Water temperature and day‐of‐year also influenced activity of both species (*P*‐values <0.001 for smoother), but at their extremes account for less than 50% of the variability, as measured by the standard deviation.

**Table 2:** Association of environmental variables with activity of (a) *M. peelii* and (b) *M. ambigua* in Broken Creek

Parameter	Estimate	Lower bound	Upper bound	Estimate as a percentage of standard deviation
(a) *M. peelii*				
Previous activity	0.295	0.294	0.296	184%
Dissolved oxygen	0.016	0.014	0.018	10%
Change in dissolved oxygen	0.066	0.048	0.084	41%
Flow	0.021	0.019	0.022	13%
Change in flow	−0.014	−0.018	−0.010	9%
Dusk compared to day	−0.004	−0.008	0.000	2%
Night compared to day	−0.002	−0.005	0.000	1%
Dawn compared to day	0.005	0.001	0.009	3%
Mean day activity	0.663	0.647	0.680	
Standard deviation (overall)	0.160	0.159	0.161	
(b) *M. ambigua*				
Previous activity	0.248	0.247	0.249	188%
Dissolved oxygen	0.011z	0.008	0.013	8%
Change in dissolved oxygen	−0.085	−0.107	−0.064	65%
Flow	0.011	0.009	0.013	8%
Change in flow	−0.004	−0.010	0.003	3%
Dusk compared to day	0.002	−0.003	0.007	1%
Night compared to day	−0.007	−0.010	−0.004	5%
Dawn compared to day	−0.006	−0.011	−0.002	5%
Mean day activity	0.723	0.699	0.748	
Standard deviation (overall)	0.131	0.130	0.132	

*Note*: Lower bound, lower 95% credible interval; upper bound, upper 95% credible interval.

## 4 DISCUSSION

We found that both *M. peelii* and *M. ambigua* in a weir pool exhibit strong site fidelity, and that *M. ambigua* used woody debris, similar to observations on riverine populations (Crook, 2004; Crook *et al*., 2001; Koehn & Nicol, 2016). In comparison, some behaviours differed from those previously observed, especially the absence of longer‐distance (tens to hundreds of kilometres) movements and a lack of habitat associations for *M. peelii*. The results also demonstrate interspecific variability in behaviours, such as disparate responses to dissolved oxygen conditions. These differences highlight the importance of understanding species‐specific fish behaviour in response to changes in environmental conditions. Overall, the results suggest that some aspects of these two native fishes' life history persist despite flow regulation, but other aspects such as longer‐distance movements may change in weir pools.

### 4.1 Habitat use

We found that habitat use of *M. ambigua* was seasonally variable, with fish more likely to be in areas with greater IWH in February (austral late summer), and lower IWH in November. Seasonal changes in use of woody debris habitats are well known for many fishes, for example in brown trout *Salmo trutta* Linnaeus 1758, European eel *Anguilla anguilla* (Linnaeus 1758) and flathead catfish *Pylodictis olivaris* (Rafinesque 1818) (Weller & Winter, 2001). While *M. ambigua* were associated with woody debris in a short‐term study (Crook *et al*., 2001) and can exhibit seasonality in habitat use (Koehn & Nicol, 2014), to our knowledge this is the first study to examine if associations with IWH vary seasonally. Woody debris can provide a range of habitat functions, including shelter, foraging sites and refuges from predators and high velocities (Crook *et al*., 2001). While the mechanism for the pattern we observed is unknown, one possible explanation could relate to spawning behaviour. Given *M. ambigua* are pelagic spawners, they may be less likely to inhabit areas with greater IWH masses during November, which is their peak spawning period in this region (Koster *et al*., 2014b, 2017). Further work is needed to explore if this is the case, and more generally to improve our understanding of the spawning dynamics of fish that use weir pools.

*M. ambigua* were also more likely to be in deeper habitats in August–December (late winter to early summer) and shallower habitats in January–February (mid to late summer). Seasonal movements between deeper and shallower habitats have also been reported for other fish species such as the congeneric Macquarie perch *M. australasica* Cuvier 1830 (Thiem *et al*., 2013) and the northern hog sucker *Hypentelium nigricans* (Lesueur 1817) (Matheney & Rabeni, 1995; Thiem *et al*., 2013). One possibility for the result observed here is that *M. ambigua* responds to changes in availability of prey. *M. ambigua* feed predominantly on macrocrustaceans such as freshwater shrimp *Paratya australiensis* (Kemp 1917) (Baumgartner, 2007), which are seasonally more abundant at warmer water temperatures, occur mostly in shallow littoral habitats (Richardson & Cook, 2006) and are abundant in Broken Creek (Reich *et al*., 2010).

We found no significant patterns of habitat use over time for *M. peelii*. This species is often associated with woody debris and deeper habitats (Jones & Stuart, 2007; Koehn, 2009; Koehn & Nicol, 2014). Seasonal differences in habitat use of *M. peelii* have also been reported, possibly related to increased water velocities, river widths and depths during higher flow periods (Koehn & Nicol, 2014). It is possible that differences between our study and these earlier ones may explain why different results were observed, such as the spatial scale at which they were conducted, the sampling locations and the methods that were used. Scales of observation, for example, are well recognized as potentially confounding estimates of habitat use (Crook *et al*., 2001; Hale *et al*., 2019; Koster & Crook, 2008). However, there are also plausible biological explanations why we may not have observed relationships, especially changes in the instream characteristics of lower Broken Creek associated with regulation, such as reduced flow variability and water level, as well as historical removal of habitat (*i.e*., fallen trees) and dredging.

### 4.2 Longitudinal movement

We found that *M. ambigua* exhibited low levels of vagility, typically moving less than 3 km from their tagging location. Other studies have reported extended periods of restricted movement for this species (Crook, 2004; Zampatti *et al*., 2018), but these are often interspersed by periods when many individuals undertake large‐scale movements (*e.g*., tens to hundreds of kilometres), often in association with high flows (Koster *et al*., 2017; O'Connor *et al*., 2005). For example, a radio‐tagging study of *M. ambigua* in the Murray River revealed that during late spring coinciding with increasing river discharge, 15 out of 19 (79%) tagged fish undertook long‐distance (>10 km) movements (O'Connor *et al*., 2005). In the current study, there were several increases in discharge (*e.g*., from 185 to 449 ml day^−1^ in September 2012, 65 to 281 ml day^−1^ in August 2013), but the water level remained relatively constant due to regulation associated with Rices Weir.

Hydraulic‐based cues such as a rise in water level rather than discharge *per se* are increasingly recognized as important to stimulating life history processes of riverine fishes such as movement (Dudley & Platania, 2007; Rakowitz *et al*., 2008). Extensive regulation and creation of weir pools throughout the MDB (Mallen‐Cooper & Zampatti, 2018) may reduce movement cues for fish, and explain the limited extent of large‐scale movements by *M. ambigua* observed here. While a study by Reynolds (1983) in the lower Murray River, which comprises extensive sections of weir pools, found some evidence of extensive movement by *M. ambigua*, this was associated with a rise in water level due to major flooding. In comparison, the changes in water level observed here may have been insufficient to elicit this response. From a conservation and management perspective, approaches such as weir pool lowering and raising to create lotic habitats upstream may be an important management strategy for *M. ambigua* and other riverine fishes that rely on flow cues (Ye *et al*., 2008).

We found that *M. peelii* movements occurred over limited spatial extents, and most often during spring (September–November). Movements were characterized by shifts away from usual locations, followed by return movements within 1–2 days, or shifts to new areas for 4–6 weeks where movement was again very limited, followed by return movements to areas previously occupied. Movements by *M. peelii* from a home location to a new position upstream, followed by a return movement to the area previously occupied during spring, have been observed in riverine and lake populations of *M. peelii* (the latter involving movements upstream into inflowing rivers) and may be spawning‐related (Koehn *et al*., 2009; Koehn & Nicol, 2016). We found that these movements were undertaken by fish larger than (or just below) 500 mm in length, the approximate size of maturity for *M. peelii* (Rowland, 1998). The similarities in the spawning season movements of *M. peelii* from contrasting habitats (*i.e*., river, lake, weir pool) suggest that some aspects of life history can continue to be supported despite flow regulation.

The mating system of *M. peelii* involves nest‐guarding and cleaning by male fish, while female fish leave the site once their eggs are deposited (Rowland, 1998). Although the sex of the fish could not be determined in our study, the return movements we observed within 1–2 days could be females visiting nest sites or males looking for mates or nesting sites. It is also possible that the shifts to new areas for 4–6 weeks could represent male nesting behaviour. Sex‐specific variation in movement linked to reproductive behaviour has also been reported for other fish species such as freshwater catfish *Tandanus tandanus* Mitchell 1838 (Koster *et al*., 2014a) and barbel *Barbus barbus* (Linnaeus 1758) (Lucas & Baras, 2001). Further tracking during the spawning season, including sex determination of tagged fish coupled with evidence of spawning (such as using larval drift nets or underwater video cameras (*e.g*., Butler & Rowland, 2009, Ebner *et al*., 2009), would be valuable for improving our understanding of the spawning‐related movement behaviour of *M. peelii*.

### 4.3 Activity patterns

*M. ambigua* were more active during periods of decreased dissolved oxygen. Fish respond to low dissolved oxygen in complex and varied ways, with increased activity potentially indicative of an avoidance response or increased swimming speed to improve gill ventilation (Chapman & McKenzie, 2009). While aquatic surface respiration has been documented for *M. ambigua* in response to hypoxia in laboratory conditions (Small *et al*., 2014), knowledge of the mechanisms *M. ambigua* use in the wild to cope with poor oxygen conditions is lacking. Determining whether the increased activity observed here relates to behavioural–physiological reactions such as increased gill ventilation or movements to avoid areas with low oxygen conditions would be valuable areas for future research. Indeed, the results of a study investigating the short‐term effects of a blackwater (*i.e*., deoxygenation) event on fish, whereby more *M. ambigua* were collected in a non‐affected site compared to a blackwater affected site (King *et al*., 2012), provide support for a hypothesis of movement of this species away (*i.e*., avoidance) from poor oxygen conditions.

In contrast to *M. ambigua*, *M. peelii* were less active during periods of decreased dissolved oxygen. In large fish such as *M. peelii* (up to 113.6 kg) (Lintermans, 2007) with high oxygen demands, decreased activity may represent an energy‐saving strategy to allow them to cope with low levels of dissolved oxygen (Crocker & Cech, 1997). It has been shown under laboratory conditions that *M. peelii* is highly sensitive to hypoxia with a swimming capacity suited to energy conservation (Small *et al*., 2014; Whiterod, 2013). These characteristics may also therefore explain why *M. peelii*, which primarily inhabits lotic riverine environments less prone to hypoxia than lentic habitats, is often disproportionately reported in hypoxic blackwater fish kills (*e.g*., less likely to escape the hypoxic zone) (King *et al*., 2012; Small *et al*., 2014).

Understanding the resultant energetic expenditure for fish when they are exposed to particular water quality conditions such as low dissolved oxygen would be a useful next step for evaluation of biological consequences (Payne *et al*., 2011; Thiem *et al*., 2018). Nonetheless, our findings demonstrate the potential consequences of altered water quality as a result of modifications such as weir pools and the importance of water quality considerations in the management of flow regimes, which often focus heavily on water volume (Poff, 2018). Importantly, when dissolved oxygen decreased to about 3 mg l^−1^, tagged *M. peelii* and *M. ambigua* were detected actively moving afterwards, demonstrating tolerance to short‐term oxygen depletion. However, if fish are exposed to longer periods of dissolved oxygen depletion, the risk of mortality may be increased (Herbert *et al*., 2011; Kramer, 1987).

We found no meaningful effect of temperature or discharge on *M. peelii* and *M. ambigua* activity. In contrast, a study of *M. peelii* in the Edwards–Wakool river system showed that both variables influenced activity of this species (Thiem *et al*., 2018). The discrepancy between the two studies could relate to differences in methodology and instream characteristics of the study systems, such as hydraulic conditions, for example lack of variation in water levels and velocity in Broken Creek. More generally, activity patterns of fishes can vary among different populations and even individuals of the same population (Metcalfe *et al*., 1999; Valdimarsson *et al*., 2000). There can be a variety of causes for this variability, including habitat, competition, and food availability and quality (Metcalfe *et al*., 1999; Metcalfe & Steele, 2001).

In conclusion, we found that some behaviours of the two focal species were similar in a weir pool to those observed in riverine populations but that there were also some considerable differences. These results highlight the potential effects of environmental change (*e.g*., flow regulation) on fish behaviours, and whether knowledge can be directly transferable among populations from different environments (Barbee *et al*., 2011). From a conservation management perspective, if fish behave differently in different habitats, management actions may need to be targeted for the specific situation. Similarly, our results demonstrate the need to understand interspecific variability in fish responses to altered environments. Collecting more information about changes in fish behaviour and life history will help determine and manage the ecological consequences of river regulation practices.

## AUTHORS CONTRIBUTIONS

W.K.: Ideas, data generation, data analysis, manuscript preparation and funding. D.D.: Data generation, manuscript preparation. A.K.: Data generation, manuscript preparation. P.M.: Data analysis, manuscript preparation. R.H.: Manuscript preparation.
